# Roll-to-Roll Production of Spider Silk Nanofiber Nonwoven Meshes Using Centrifugal Electrospinning for Filtration Applications

**DOI:** 10.3390/molecules25235540

**Published:** 2020-11-26

**Authors:** Fabian Müller, Shakir Zainuddin, Thomas Scheibel

**Affiliations:** 1 Lehrstuhl Biomaterialien, Universität Bayreuth, Prof-Rüdiger-Bormann-Str.1, 95447 Bayreuth, Germany; fabian.mueller@bm.uni-bayreuth.de (F.M.); shakir.zainuddin@bm.uni-bayreuth.de (S.Z.); 2 Bayreuther Zentrum für Kolloide und Grenzflächen (BZKG), Naturwissenschaften I, Universität Bayreuth, Universitätsstr. 30, 95440 Bayreuth, Germany; 3 Bayreuther Materialzentrum (BayMat), Fakultät für Ingenieurswissenschaften, Universität Bayreuth, Universitätsstr. 30, 95440 Bayreuth, Germany; 4 Bayreuther Zentrum für Molekulare Biowissenschaften (BZMB), Universität Bayreuth, Naturwissenschaften I, Universitätsstr. 30, 95440 Bayreuth, Germany; 5 Bayrisches Polymerinstitut (BPI), Universität Bayreuth, Universitätsstr. 30, 95440 Bayreuth, Germany

**Keywords:** nanomaterials, spinning technology, nonwoven fabrics, spider silk, filter technology

## Abstract

Filtration systems used in technical and medical applications require components for fine particle deep filtration to be highly efficient and at the same time air permeable. In high efficiency filters, nonwoven meshes, which show increased performance based on small fiber diameters (e.g., using nanofibers), can be used as fine particle filter layers. Nanofiber nonwoven meshes made by electrospinning of spider silk proteins have been recently shown to exhibit required filter properties. Needle-based electrospinning, however, is limited regarding its productivity and scalability. Centrifugal electrospinning, in contrast, has been shown to allow manufacturing of ultrathin polymer nonwoven meshes in an efficient and scalable manner. Here, continuous roll-to-roll production of nonwoven meshes made of recombinant spider silk proteins is established using centrifugal electrospinning. The produced spider silk nanofiber meshes show high filter efficiency in the case of fine particulate matter below 2.5 µm (PM2.5) and a low pressure drop, resulting in excellent filter quality.

## 1. Introduction

There is an increasing demand for high-quality air filters for various applications, e.g., ventilation and air conditioning systems for cleanrooms, laboratories, real estate, automotive and aircraft industries, air-treatment for medical use, and protective face masks. Such demand has even been increased upon recent developments concerning virus removal from air in the COVID-19 pandemic. Efficient and highly permeable fine particle filter materials, in particular, are in the focus of research for deep filtration [1,2]. Electrospun sub-micron and nanofiber nonwoven meshes offer great filtration efficiency for small particulate matter (<2.5 µm (PM2.5)) in combination with high air permeability, resulting in excellent filter qualities [1,2,3]. Application of such nonwoven meshes improves the performance of filtration systems by increasing filtration capacity and lowering flow resistance [4,5]. However, the weak mechanical performance of ultrathin fibers combined with their increased interaction with other materials due to their large surface is challenging in production and handling. Further, it is envisioned to use nontoxic and biodegradable materials for ultrathin fibers to avoid putative health issues, in case fibers or particles are set free and inhaled [5]. 

Spider silk fibers show unique properties, such as the combination of versatile strength and extensibility, in addition to biocompatibility [6,7,8]. Since spider silk materials are mechanically robust, biodegradable, and neither the silks nor their metabolites are toxic or cause allergic reactions when incorporated in the human body, they are a promising sustainable candidate for sub-micron and nanofibrous filter applications [9,10,11,12]. Electrospinning (ES) is one versatile technique to produce sub-micron- and nanofibers from natural [13,14], artificial [11,12], and synthetic polymers [15,16]. Spider silk fibers produced by ES can be collected as a nonwoven mesh or as aligned fibers according to their desired applications [12,17]. The fiber diameter can be adjusted by varying the electric field strength and the concentration of the spinning solution. As one drawback, ES shows only relatively low productivity (about 0.1 g·h^−1^ per nozzle). Facing this challenge with needle-based ES, the emphasis of scientific research is increasingly shifted towards nozzle-free spinning techniques like rotating roller electrospinning [18], wire electrode electrospinning [19], and centrifugal spinning (CS) [20,21].

Recently, centrifugal electrospinning (CES) has been introduced for spinning of polymeric nonwoven meshes with fiber diameters at the scale of nanometers with significantly higher productivity compared to that of needle-based ES [22,23]. The used CES device contains a rotatable spinning bell as an electrode in which the spinning solution can be injected. Similar to centrifugal spinning, the rotating spinneret drives the injected spinning solution towards the rim of the bell. As soon as the centrifugal force overcomes the surface tension of the spinning solution, the rotating bell enables simultaneous formation of a multitude of fibers along its rim, thus increasing productivity. An applied electric field perpendicular to the rotational plane is used to further focus and elongate the occurring fibers. A metal plate used as a counter electrode is acting as a collector. Furthermore, a compressed air stream is used along the spinning bell to additionally stretch and dry the forming fibers. These three parameters allow perfect control of the particular process and spinning parameters and make the process more adjustable, enabling a highly controllable and reproducible method for production of nanofibers. 

In this study, nonwoven nanofiber meshes were made of previously established recombinant spider silk protein eADF4(C16) using a novel roll-to-roll CES process, and the applicability of the resulting nonwoven meshes was shown in a fine dust filtration setup.

## 2. Results and Discussion

### 2.1. Centrifugal Electrospinning of Spider Silk Proteins

In CES, both the rotational speed of the spinning bell and applied airflow have an effect on fiber formation and deposition. In order to determine suitable spinning conditions for the recombinant spider silk protein eADF4(C16), the protein concentration, process flowrate, electric field strength, and rotational speed have been analyzed individually. 

Due to rotation of the bell and turbulences in CES in combination with the multitude of jets occurring at the same time, the nonwoven meshes showed a high degree of interconnection, e.g., bending, twisting, and knotting. Nonetheless, the nonwoven meshes formed homogeneous layers made of randomly oriented fibers without holes or damages.

**Figure 1:**
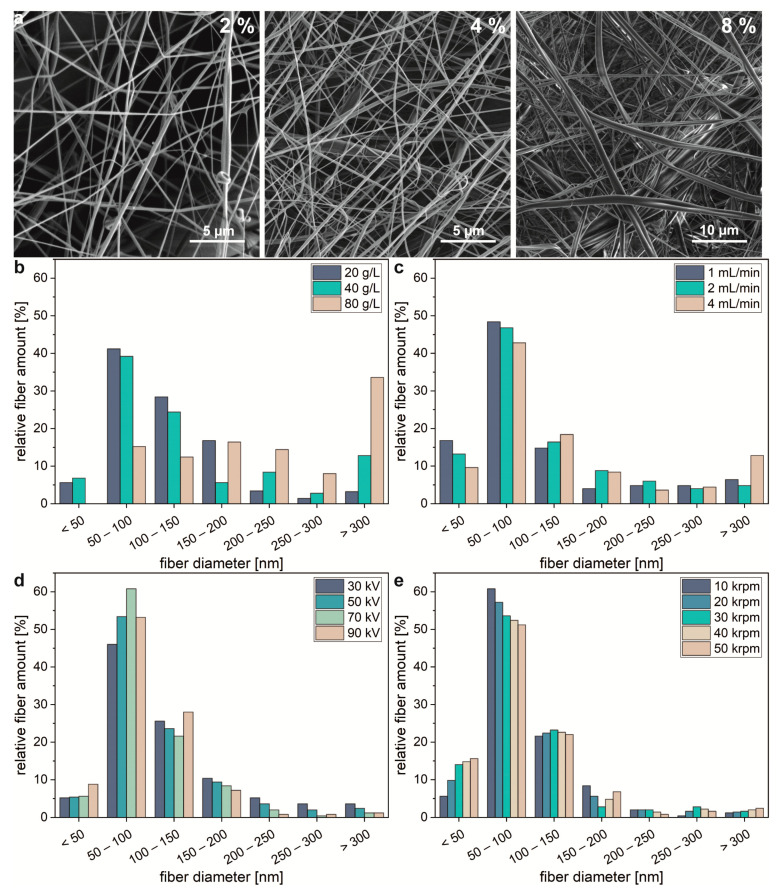
Nonwoven fiber diameter dependence on process parameters. (**a**) SEM images of centrifugal electrospun spider silk nonwoven meshes spun out of solutions at different concentrations given in % (*w/v*). (**b**) Fiber diameter distribution of spider silk nonwoven meshes made at different protein concentrations, (**c**) volumetric flow rates, (**d**) voltages, and (**e**) rotational speeds. For each experimental group, 250 independent samples (*n* = 250) were tested. Further spinning parameters were (**a**,**b**) Q = 8 mL/min, U = 90 kV, and ω = 10 krpm; (**c**) c = 2% (*w/v*), U = 90 kV, and ω = 10 krpm; (**d**) c = 2% (*w/v*), Q = 2 mL/min, and ω = 10 krpm; (**e**) c = 2% (*w/v*), Q = 2 mL/min, and U = 70 kV. The potential of the collector was −5 kV in all measurements.

The viscosity of the spinning solution, which describes its resistance to deformation, depended on temperature, protein concentration, and the solvent. To analyze the impact of the protein concentration and to investigate appropriate spinning conditions, silk meshes were spun out of spider silk solutions at 2%, 4%, and 8% (*w/v*). The flow rate was Q = 8 mL/min, the voltage, U = 90 kV, and the rotational speed, ω = 30 krpm. An increasing concentration resulted in thicker fiber diameters (Figure 1a). The average fiber diameter increased from 122 ± 5 to 295 ± 15 nm for spinning solutions at concentrations of 1% and 8% (*w/v*), respectively (Figure 1b). 

Increasing the process temperature would promote evaporation of the solvent and drying of the meshes. Since the vapor pressure of hexafluoroisopropanol (HFIP) is 160 hPa (20 °C) and eADF4(C16) is not meltable, experiments were carried out at room temperature.

In addition to the concentration of the spinning solution, flow rate was of huge importance. To determine the effect of flow rate on fiber diameter and morphology of the formed mesh, experiments with flow rates of 1, 2, and 4 mL/min were carried out. The concentration was c = 2% (*w/v*), the voltage, U = 90 kV, and the rotational speed, ω = 30 krpm. Upon increasing the flow rate, the fiber diameters increased (Figure 1c). The average fiber diameter was 109 ± 5, 123 ± 7, and 170 ± 14 nm for volumetric flow rates of 1, 2, and 4 mL/min, respectively. 

As with needle-based electrospinning (ES) [24,25,26], an increasing electric potential at the spinneret increased the charge of the occurring jets and, thereby, enhanced repulsion from the spinning bell and whipping instabilities, resulting in elongation of the jets and lowering of their diameters [23]. The oppositely charged collector (−5 kV) additionally attracted occurring fibers. Further, an increased electric field strength increased the mass flow from the spinneret, which increased the resulting fiber diameters [27]. Therefore, higher electric field strengths resulted to a certain extent in thinner fibers in the collected nonwoven meshes. To demonstrate the impact of the electric field strength on fiber diameters, silk meshes were spun at different applied voltages. The other parameters were c = 2% (*w/v*), Q = 2 mL/min, and ω = 10 krpm. Upon varying the potential at the spinneret from 30 to 90 kV, the fibers in the collected nonwoven mesh became thinner in diameter and denser (Figure 1d). The average fiber diameter was 125 ± 5, 96 ± 4, 95 ± 3, and 96 ± 4 nm for electric potentials of 30, 50, 70, and 90 kV, respectively.

In CES, in addition to electric potential, the rotation of the spinning bell drives the solution to overcome its surface tension, and due to their inertia the centrifugal force stretches occurring jets [23]. To evaluate the effect of the rotational speed on mesh formation and morphology of the produced fibers, experiments at different rotational speeds were carried out at c = 2% (*w/v*), Q = 2 mL/min, and ω = 70 kV. Upon varying the rotational speed of the spinneret from 10 to 50 krpm, the morphology of the produced spider silk nonwoven mesh varied marginally. Increasing the rotational speed resulted in slightly thinner fiber diameters (Figure 1e), but also resulted in more twisted and entangled fibers. The average fiber diameters were 97 ± 3, 96 ± 3, 95 ± 4, 94 ± 4, and 95 ± 4 nm for rotational speeds of 10, 20, 30, 40, and 50 krpm, respectively. 

### 2.2. Effect of CES on β-Sheet Content in as Spun Spider Silk Fibers

The secondary structure of spider silk fibers is important for their mechanical and physico-chemical properties [28,29]. High values of β-sheet content indicate high crystallinity, which is one basis for the mechanical properties of native spider silk fibers, i.e., β-sheet content dictates fiber strength and water insolubility [12,28]. Spider silk fibers with low crystallinity can be stabilized by subsequent post-treatment inducing β-sheet structures; however, it is desirable to have an initial high β-sheet content already in as-spun fibers. To compare the as-spun and post-treated β-sheet content, meshes produced using needle-based ES and CES were analyzed in both states. For post-treatment, all samples were treated with ethanol vapor to preserve the morphology of the spider silk nonwoven meshes as published previously [12].

**Figure 2:**
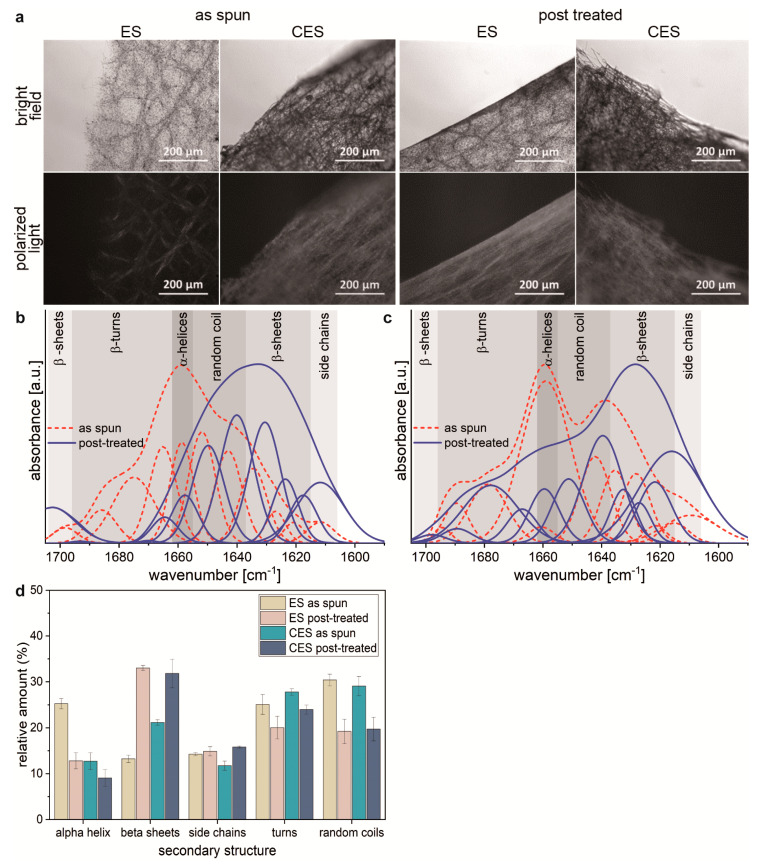
Effect of spinning method on β-sheet content of produced spider silk fibers. (**a**) Comparison of bright field and birefringence images of needle electrostatic spun (ES) and centrifugal electrospun (CES) spider silk fiber meshes as-spun and after ethanol vapor treatment (post-treatment). As-spun CES fibers showed birefringence even without post-treatment. Comparison of normalized FTIR spectra including deconvolution of needle electrospun (**b**) and centrifugal electrospun (**c**) silk protein fiber meshes as spun and after ethanol vapor post-treatment. (**d**) Secondary structure elements as determined using FTIR analysis of needle electrospun (ES) and centrifugal electrospun (CES) silk protein fiber meshes before (as-spun) and after post-treatment.

Highly ordered noncubic crystalline structures frequently have the feature that their refractive index is dependent on polarization and propagation of light passing through them, which is called birefringence [30,31]. Therefore, these structures are able to split a light beam in a parallel and perpendicular polarized component [32]. When polarized light is passing, in samples containing such highly ordered crystalline structures the light is refracted, which can be displayed by using a second polarization filter. Light not refracted by the sample cannot pass the second polarization filter in contrast to refracted light. Hence, crystalline structures in the analyzed sample appear bright in contrast to amorphous structures, which appear shaded. Therefore, birefringence can be used to analyze proteinous materials [33,34,35], like spider silk fibers, concerning their crystallinity and crystal orientation [29,31]. Birefringence analysis of the needle electrospun spider silk nonwoven meshes showed almost no signals, whereas the centrifugal electrospun mesh and post-treated samples showed birefringence, indicating higher β-sheet content and structural alignment (Figure 2a). 

Fourier transform infrared spectroscopy (FTIR) was used to further examine the secondary structure content of the fibers by analyzing the amide I band (Figure 2b,c). Samples with α-helical content, typical for silk spun out of HFIP, showed a maximum around 1658 cm^−1^, whereas β-sheet-rich samples showed a peak at 1622 cm^−1^. ES spider silk nonwoven meshes exhibited a peak at 1658 cm^−1^, indicating high α-helical content. After post-treatment, the peak shifted to 1631 cm^−1^, verifying the successful structural transformation into β-sheets. Interestingly, as-spun fibers using centrifugal electrospinning showed an increased β-sheet and β-turn content. 

Fourier self-deconvolution (FSD) was used to analyze the secondary structure composition in more detail (Figure 2d). As-spun ES spider silk protein nonwoven fibers exhibited a content of α-helical (25%) and random coil (30%) and a lower amount of β-sheet structures (13%), which increased to 33% upon post-treatment at the expense of α-helical (13%) and random coil content (19%). Side chains and turns remained similar in both samples. In contrast, nonwoven meshes produced using CES revealed 21% of β-sheet structures directly after spinning and comprised only 13% of α-helical structures. β-sheet content increased to 32% after post-treatment. The initial higher β-sheet content of samples spun using CES might be a result of enhanced shearing of the proteins during the CES process, which is known to be involved in structure formation during the natural spinning process too [23]. Since spider silk nonwoven meshes produced using CES were already showing sufficient crystallinity and stability compared to those produced using needle electrospinning, post-treatment was not necessary, providing the basis to develop roll-to-roll nonwoven mesh production.

### 2.3. Roll-to-Roll Nonwoven Mesh Production

**Figure 3:**
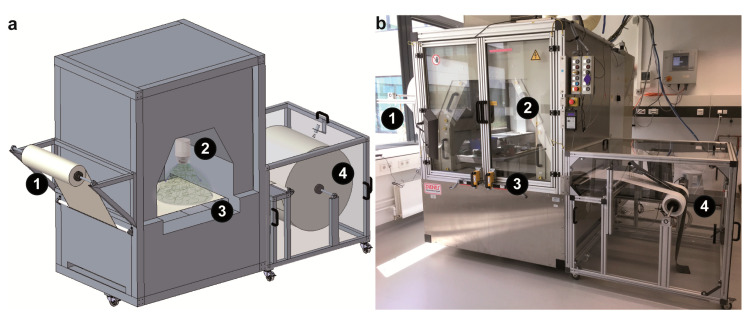
Continuous production of spider silk nanofiber meshes in a roll-to-roll process. (**a**) Model and (**b**) photo of polyamide support bracket (1) and winding unit (4) mounted at the centrifugal electrospinning device. The polyamide (PA) support is dragged by a step motor connected to the winding shaft (4) through the spinning chamber and over the collector (3), where it is coated with nanofibers by the spinneret (2).

In general, polymer throughput of a spinning technique depends on the chosen concentration of the spinning solution and the configured flow rate. In this context, productivity of CES is around a thousand times higher compared to that of single needle electrospinning [23]. Significantly increased productivity makes CES suitable for continuous nanofiber production, and therefore a roll-to-roll process was developed using slits on both sides of the spinning chamber (Figure 3). To ensure constant pulling speed, unwinding and rewinding of the roll was automatically realized using a motor, which was controlled by an ultrasonic sensor measuring the roll diameter and adjusting the winding speed, accordingly. Due to its highly electrostatic charge when leaving the CES device, the material attracted dust particles from its surroundings, which could be observed in SEM images. Therefore, polycarbonate panes were installed to protect the produced meshes from dust. 

**Figure 4:**
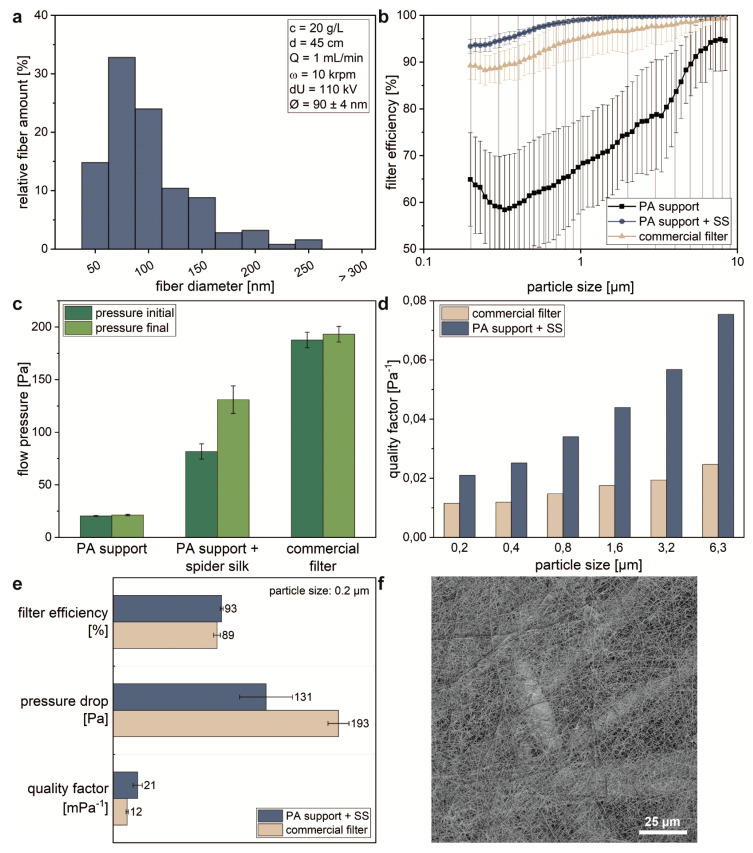
Characterization of spider silk nonwoven meshes as filter material. (**a**) The fiber diameter distribution shows that the majority of the silk fibers in the filter are nanofibers (i.e., <100 nm in diameter); 250 independent samples (*n* = 250) were tested. (**b**) Filter efficiency and (**c**) air drag comparisons of polyamide (PA) support, spider silk-covered PA support, and a commercial three-layer dust filter. For each experimental group, 10 independent samples (*n* = 10) were tested. (**d**) Quality factor of spider silk containing filter setup in comparison to a commercial filter. (**e**) Filter characteristics of uncovered polyamide support, spider silk (SS)-covered PA support, and the commercial filter determined using 0.2 µm particles. (**f**) SEM image of spider silk-covered PA support used as particle filter before filtration.

The morphology of spider silk nonwoven meshes produced in the continuous roll-to-roll process was indistinguishable to that produced in the batch setup (Figure 4f). Concerning the SEM images, morphology and distribution of the meshes were homogeneous, and the mesh was not affected by the rolling process. Additionally, storage for months, handling, and shipping did not affect the meshes’ appearance. Therefore, as-spun meshes could be directly used for filter efficiency tests. 

### 2.4. Application of CES-Produced Spider Silk Meshes as Fine Dust Filters

ES spider silk nanofibers have already been applied in fine dust filter systems showing better quality factors in comparison to commercially available filters. The quality factor is determined by the filtration efficiency of a filter, which describes the ability to filter a certain contaminant (e.g., particles with defined diameters), and its permeability for the medium (air, fuel, and oil) [11]. The produced CES spider silk mesh on the polyamide (PA) substrate showed a fiber diameter distribution (Figure 4a) with a majority (72%) of nanofibers (with diameters below 100 nm) and an average fiber diameter of 90 ± 3 nm. The slightly reduced fiber diameters in the roll-to-roll setup in comparison to the batch setup are a result of increased negative potential at the collector in the roll-to-roll process. The fibers covered the PA support homogeneously, forming a consistent PA/spider silk mesh bond (Figure 4f). To investigate the filtration efficiency of the continuously produced spider silk nonwoven mesh, filtration tests of the PA substrate, spider silk/PA bond, and a commercial filter were performed (Figure 4b). With decreasing particle size, the PA substrate showed a decreasing filtration efficiency, reaching its minimum of 58% at 0.33 µm. The commercial filter exhibited a distinct higher filtration efficiency for all particle sizes. It showed a minimum of 88% efficiency at 0.25 µm. The filtration performance of the spider silk/PA bond showed the best performance, and the lowest filtration efficiency was determined to be 94% for particles sizes of 0.2 µm. This was in accordance with previous results obtained using needle-based ES in a static setup [11].

The single PA support showed a high air permeability indicated by the low flow pressure before (20 Pa) and after (21 Pa) the filtration tests (Figure 4c). As expected, the bond of PA support and spider silk mesh showed a lower permeability with flow pressures of 81 Pa before and 131 Pa after the filtration tests. These flow pressures were significantly lower than those of the commercial filter, which showed a flow pressure of 188 Pa before and 193 Pa after filter tests. 

As filtration efficiency depends on pressure loss, and therefore includes energy uptake, the quality factor Equation (1) represents one important parameter for evaluation of filter materials [11]. Quality factors were calculated for each given particle size using the filter efficiency and flow pressure from 10 independent measurements. The spider silk-based filter material showed a significantly higher filtration quality factor compared to the commercial filter material for all particle sizes (Figure 4d).

In the dry state, there is no indication of degradation of spider silk meshes at all, since the protein-based meshes typically degrade enzymatically, which takes place in liquids. Concerning mechanical robustness, the fine filtration layer is embedded and thereby protected by the other filtration layers, and the support material acts as a capping layer after assembly of the filter. 

## 3. Materials and Methods 

### 3.1. Centrifugal Electrospinning of Recombinant Spider Silk Proteins

A recently developed CES setup [23] (DIENES Apparatebau, Mühlheim am Main, Germany) was used to produce nonwoven meshes made of recombinant spider silk protein eADF4(C16) (AMSilk, Planegg/München, Germany) out of hexafluoroisopropanol (HFIP; ABCR, Karlsruhe, Germany). eADF4(C16) is an engineered 48 kDa fibroin-4 silk protein derived from the dragline of the European garden cross spider (*Araneus diadematus*). It’s made of 16 repeats of the consensus sequence (C-module: GSSAA AAAAA ASGPG GYGPE NQGPS GPGGY GPGGP) found in the repetitive core domain of the natural fibroin-4 silk protein. The eADF4(C16) solution was injected through a hose line into the rotatable spinning bell using a syringe pump. The solutions with concentrations of 10, 40 and 80 g/L were loaded into 50 mL syringes and pumped into the rotating center bell at a rate of 1–8 mL/min. The spinning bell was driven by a turbine, and its speed was controlled by an optical sensor. The maximum speed was 50 krpm resulting in a relative centrifugal force of 70,000 G at the rim of the spinning bell. A guiding air stream (0–6 bar) sheathed the rotating bell pointing towards the collector. Using two individual high voltage supplies, the electric potential could be adjusted between 0 and +90 kV for the bell and 0 and −20 kV for the collector, depending on the intended fiber diameter. To collect the spider silk meshes, a nonconductive polyamide (PA) microfilament nonwoven fabric was placed on a flat metal collector (40 × 40 cm) covering it completely. The PA fabric acted as a support, enabling further handling of the spider silk nonwoven meshes. A large nonconductive spinning chamber with exhaust suction surrounded by a grounded metal casing (DIENES Apparatebau, Mühlheim am Main, Germany), in which the spinneret and the collector were placed, was used to secure the spinning process. A roll-to-roll process was set up using suspensions for supply, take-up spindles for the support material, and a motor to pull the support through the CES device. To enable a constant pulling speed of the support material, the motor was controlled by an ultrasonic sensor measuring the diameter of the take-up spindle and adjusting the winding speed, accordingly. During roll-to-roll production, the potential at the collector was increased (−20 kV) because the support dragged charges from the collector and thereby lowered its potential. 

### 3.2. Post-Treatment of Spider Silk Nonwoven Meshes

Nonwoven meshes produced in batch setups were placed hanging freely inside a sealable vessel according to a previously established protocol [12]. The vessel was filled with 50 mL of ethanol from the bottom using a syringe attached to a tubing system. Utilizing a heater, 60 °C was induced and held for 120 min. After 10 min of cooling at room temperature (RT), ethanol was removed from the vessel using the respective syringe. The procedure was repeated with water, which was filled into the vessel through an analogous tubing system [12]. Since as-spun spider silk nonwoven meshes produced using CES showed sufficient β-sheet contents and appeared to be robust, no further post-treatment was necessary, which was important for establishing roll-to-roll nonwoven mesh production.

### 3.3. Secondary Structure Characterization Using FTIR 

Secondary structures of batch-produced as-spun and post-treated spider silk meshes were analyzed using FTIR spectroscopy (Tensor I with IR Microscope Hyperion 3000; Bruker, Billerica, MA, USA) and subsequent Fourier self-deconvolution (FSD) [36]. For each spectrum, 60 scans were accumulated and averaged in transmittance mode at wavenumbers ranging from 800 to 4000 cm^−1^. A curve fit was performed for quantitative analysis of the constituent bands within the amide I band between 1590 and 1705 cm^−1^. The process included baseline correction and a local least square fit to analyze single contribution peaks according to the corresponding peak positions as previously published (1611, 1619, 1624, 1630, 1640, 1650, 1659, 1666, 1680, 1691, and 1698 cm^−1^) [36].

### 3.4. Scanning Electron Microscopy of Spider Silk Meshes 

Round samples with a diameter of 10 mm were produced and sputter coated with 1.3 nm platinum prior to imaging using scanning electron microscopy (Sigma 300 VP, Zeiss, Oberkochen, Germany) at a voltage of 2 kV. Fiber diameters were analyzed using ImageJ software. The fiber diameter distribution was calculated based on 250 individual fibers each. For all diameter measurements depicted from SEM images, the platinum layer from the sputter coating on the fiber’s surface was not taken into account. Therefore, the actual diameter of spider silk fibers was slightly lower.

### 3.5. Birefringence Analysis

Brightfield and polarized light microscopy (PLM) were carried out using a stereo microscope (DMI3000B; Leica Microsystems, Wetzlar, Germany). Light sources were in the visible range (halogen lamp, 100 W, 2900 K). To obtain brightfield images, samples were illuminated and observed from beneath. Regarding PLM, the light was primarily polarized at 90° using a first polarizing lens, subsequently passing a second polarizing filter (analyzer). 

### 3.6. Quantification of Filter Efficiency and Quality Factor

The filter efficiency was obtained using an MFP 2000 (Palas, Karlsruhe, Germany). Then 300 mg/m^3^ of ISO 12103-1 A2 fine test dust (PTI Powder Technology, Arden Hills, MN, USA) with particle sizes ranging from 0.2 to 8.9 µm was blown through a pipe with a 28.3 cm^2^ cross-section for 30 s with air flow at a flow rate of 42.5 L/min and a velocity of 25 cm/s. The number of dust particles passing the pipe was measured with and without a filter. The particle count was carried out using a built-in light scattering spectrometer. In comparison, filter material of a conventional dust filter bag (MicrofiltPlus, AE120, AEG, Frankfurt am Main, Germany) was used. Ten independent samples were tested for each filter. The quality factor (QF) was calculated using the measured particle deposition efficiency (PD) and pressure drop (∆p) for the given particle sizes in Equation (1) [11].

QF = ln[(PD^−1^)]·∆p^−1^, (1)

## 4. Conclusions

Using centrifugal electrospinning, a roll-to-roll process was established for producing ultrathin nonwoven meshes of recombinant spider silk applicable in fine dust filter systems. The advanced CES device combines an electrical and a centrifugal field with an air stream, enabling efficient production of spider silk nanofibers at a production rate that is up to 1000 times higher than that of traditional spinning methods. Fibers spun using this CES technique showed a significantly higher β-sheet content than those spun using needle-based electrospinning, indicating greater mechanical and chemical stability. Therefore, no post-treatment was necessary, which allowed development of a roll-to-roll process. Fine dust filtration tests of spider silk meshes produced with such a setup revealed filter efficiencies (PM2.5) and quality factors significantly higher than those of a commercial filter system, opening the road towards applications in scale-up processes.
